# Determinants of Population-Based Cancer Screening Performance at Primary Healthcare Institutions in China

**DOI:** 10.3390/ijerph18063312

**Published:** 2021-03-23

**Authors:** Senshuang Zheng, Xiaorui Zhang, Marcel J. W. Greuter, Geertruida H. de Bock, Wenli Lu

**Affiliations:** 1 Department of Epidemiology, University Medical Center Groningen, University of Groningen, 9713 CP Groningen, The Netherlands; s.zheng02@umcg.nl (S.Z.); g.h.de.bock@umcg.nl (G.H.d.B.); 2 Department of Epidemiology and Health statistics, Tianjin Medical University, Tianjin 300070, China; TMUxiaorui@163.com; 3 Department of Radiology, University Medical Center Groningen, University of Groningen, 9713 CP Groningen, The Netherlands; m.j.w.greuter@umcg.nl; 4 Technical Medical Centre, Robotics and Mechatronics (RaM) Group, Faculty of Electrical Engineering Mathematics and Computer Science, University of Twente, 7522 NH Enschede, The Netherlands

**Keywords:** breast cancer, cervical cancer, colorectal cancer, cancer screening, community participation, primary healthcare

## Abstract

Background: For a decade, most population-based cancer screenings in China are performed by primary healthcare institutions. To assess the determinants of performance of primary healthcare institutions in population-based breast, cervical, and colorectal cancer screening in China. Methods: A total of 262 primary healthcare institutions in Tianjin participated in a survey on cancer screening. The survey consisted of questions on screening tests, the number of staff members and training, the introduction of the screening programs to residents, the invitation of residents, and the number of performed screenings per year. Logistic regression models were used to analyze the determinants of performance of an institution to fulfil the target number of screenings. Results: In 58% and 61% of the institutions between three and nine staff members were dedicated to breast and cervical cancer screening, respectively, whereas in 71% of the institutions ≥10 staff members were dedicated to colorectal cancer screening. On average 60% of institutions fulfilled the target number of breast and cervical cancer screenings, whereas 93% fulfilled the target number for colorectal cancer screening. The determinants of performance were rural districts for breast (OR = 5.16 (95%CI: 2.51–10.63)) and cervical (OR = 4.17 (95%CI: 2.14–8.11)) cancer screenings, and ≥3 staff members dedicated to cervical cancer screening (OR = 2.34 (95%CI: 1.09–5.01)). Conclusions: Primary healthcare institutions in China perform better in colorectal than in breast and cervical cancer screening, and institutions in rural districts perform better than institutions in urban districts. Increasing the number of staff members on breast and cervical cancer screening could improve the performance of population-based cancer screening.

## 1. Introduction

Cancer is one of the leading causes of death and a large public health issue in China [1,2]. The age-standardized incidence and mortality rate of cancer were 186 and 106 per 100,000 in 2015, respectively [3], and the proportion of disability-adjusted life years caused by cancer has risen from 14.6% to 17.1% during the last decade [4]. The World Health Organization recommends that breast, cervical, and colorectal cancers are suitable for screening, and are curable if detected and treated early [5]. A high population coverage and adherence rate are critical factors for a successful screening program, but achieving these goals is challenging for many countries [6,7]. Previous global studies observed that the coverage rate for cervical cancer screening was on average 40% (range 1–80%) in 57 countries, and 19% (range 1–73%) in developing countries, and an overall adherence rate of 33% for colorectal cancer screening in developed countries [8,9]. In a study from China uptake rates under 27% were reported for breast, cervical, and colorectal cancer screenings [10,11].

Participation of cancer screening may be affected by several factors [12]. Prior studies have noted the crucial role of organizational measures for screening performance and prompting participants to adopt cancer screening [12,13,14,15,16,17,18]. Sequist et al., Page et al., and Püschel et al. identified that sending invitations by mail, and telephone call reminders to residents effectively increased screening participation [18,19,20]. Mao et al. indicated that improving the willingness of institutions to provide screening services and providing stable funding for programs would ensure screening performance [16]. Several studies have shown a positive effect of simplifying the cervical and colorectal cancer screening test [21,22,23].

In China, most of the population-based cancer screening programs are provided by primary healthcare institutions. Given the current low coverage and adherence rates of cancer screening, this study aims to determine the organizational factors that influence the performance of population-based cancer screening in primary healthcare institutions. This may help screening program organizers and healthcare providers to improve the performance of cancer screening.

## 2. Materials and Methods

### 2.1. Context

The performance of population-based cancer screening was assessed for breast, cervical, and colorectal cancer screening in primary healthcare institutions in Tianjin, China. Primary healthcare institutions received 54.1% (4.43 billion) of all outpatient visits and 18.2% (44.5 million) of all hospitalized patients nationwide in 2017, and therefore play an important role in providing healthcare to urban and rural residents [2]. In addition to basic medical and basic public health services, the primary healthcare institutions also provide fee free cancer screening service paid by the Chinese government [24]. These institutions send invitations to the target population, perform the tests, communicate the results, and perform the follow-up of screening [25]. In order to increase participation the Chinese government determined a target number of screenings performed per institution per year based on the regional population. The actual number of screenings was compared to these target numbers.

### 2.2. Study Design and Participants

We carried out a cross-sectional study of cancer screening performance at primary healthcare institutions. Data including characteristics of institution, medical staff dedicated to screening, and implementation of the screening process were collected to identify the factors that influence the screening performance.

We applied a survey in all primary healthcare institutions in Tianjin. There are 262 primary healthcare institutions in 16 districts, with 95 institutions in urban districts and 167 institutions in rural districts.

### 2.3. Questionnaire

Each institution was sent a questionnaire which was answered for breast, cervical, and colorectal cancer screening separately (Table 1). Face and content validity of the questionnaire were assessed by 11 independent professionals in cancer screening and primary healthcare.

Questionnaires were collected by trained investigators at these institutions. To provide sufficient time for filling in the questionnaires and collecting them all from all over the large geographical area of Tianjin, all data were collected between November 2017 and May 2018.

We determined completion of cancer screening as the dependent variable. Completion of cancer screening means that the actual number of screenings provided by the institution is greater than or equal to the target number determined by the government per year based on the regional population. Urban district, GDP, number of screening tests, frequency of staff training, number of staff dedicated to screening, frequency of introduction to residents of screening programs, and telephone invitation of the target population that may have influenced the completion of the cancer screening were the determinants for exploration.

### 2.4. Ethical Considerations

The questionnaire did not include private information, and the heads of institutions were provided information on the purpose of the study and provided verbal consent before the questionnaire survey. This study was approved by the Medical Ethics Committees of Tianjin Medical University, as it is part of “A study of breast cancer screening strategy in Chinese community based on system dynamic and Hopfield neural network model”, which is founded by the National Natural Science Foundation (No. 72074166).

### 2.5. Statistical Analysis

Descriptive statistics were applied, including median and inter-quartile range (IQR) for continuous variables, and frequencies for categorical variables. To make the grouping of GDP balanced, we used the average GDP of 15 districts of Tianjin (¥80 billion) excluding the relatively high GDP of Binhai New Area as grouping basis. Univariate logistic regression analyses were used to analyze which factors determined that institutions completed the target number of breast, cervical, and colorectal cancer screenings. Additionally, univariate analyses stratified by urban and rural district were conducted. Variables with significance less than 0.2 in the univariate logistic regression analyses were included in the multivariate logistic regression models. The number of staff dedicated to screening was categorized as ≥ 3 staff members versus less for breast and cervical cancer screening, and ≥ 10 staff members versus less for colorectal cancer screening. Odds ratios and 95% confidence intervals (CI) were estimated. A *p*-value of < 0.05 was considered as a significant difference. Statistical analysis was performed using IBM SPSS Statistics, version 23.0 for Windows.

## 3. Results

All 262 (100%) primary healthcare institutions responded to the questionnaire. There were 206 (78.6%), 231 (88.2%), and 261 (99.6%) institutions answering the questions of breast, cervical, and colorectal cancer screenings, respectively. Missing data due to incomplete questionnaires were not included in the analysis. The characteristics, screening investment and performance of institutions are shown in Table 2.

### 3.1. Characteristics of Institutions

For breast cancer screening all institutions (100%) performed clinical breast examinations (CBE), 36.1% ultrasound, but none (0%) mammography. For cervical cancer screening 95.6% of the institutions had a gynecological examination table and 52.2% of the institutions performed pap smear observations. For colorectal cancer screening all institutions (100%) performed fecal immunochemical tests (FIT). In 58% and 61% of the institutions between 3 and 9 staff members were dedicated to breast and cervical cancer screening, respectively; in 71% of the institutions ≥10 staff members were dedicated to colorectal cancer screening (Table 2).

### 3.2. Cancer Screening Performances

Over 90% of the institutions performed more than the target number of colorectal cancer screening, while 60.5% and 58.6% performed more than the target number of breast and cervical cancer screenings. Less than 2000 screenings per year were performed in 71.6% and 69.5% of the institutions for breast and cervical cancer screening, respectively; more than 2000 screenings were performed in 53.6% of the institutions for colorectal cancer screening. The median referral rates were 1.2, 1.3, and 2.2 for breast, cervical, and colorectal cancer screenings, respectively (Table 2).

### 3.3. Factors Influencing the Completion of Cancer Screening

In the univariate analysis it was found that primary healthcare institutions in rural districts were more likely to complete cancer screening than institutions in urban districts (breast: 78.7% vs. 37.0%, cervical: 73.4% vs. 33.8%, colorectal: 96.0% vs. 88.9%). Institutions in districts with GDP > ¥80 billion performed worse completion rate of the target number in breast and cervical cancer screenings than those in low GDP districts (breast: 69.5% vs. 51.8%, cervical: 66.1% vs. 49.4%). This difference was not observed for the completion of the target number in colorectal cancer screening. Having over three employed persons dedicated to breast and cervical cancer screening did increase the completion of performing more than the target number (breast: 66.1% vs. 46.7%, cervical: 64.5% vs. 40.0%). No significant differences were found in the number of screening tests applied and the frequency of staff training. Interestingly, inviting residents by telephone and introduction to residents of the screening programs did not show a positive effect (Table 3). Furthermore, in rural institutions, there was no statistical difference in the completion of the target number among each variable grouping. In urban institutions, having more than 3 employed persons dedicated to colorectal cancer screening contributed to completing the target number. (Supplementary Materials Tables S1 and S2).

Considerable differences were evident in the completion according to the districts of institutions for breast and cervical cancer screenings in the multivariate regression models. Significantly more institutions in rural districts fulfil the target number of screenings performed (breast: OR = 5.16 (95%CI: 2.51–10.63), cervical: OR = 4.17 (95%CI: 2.14–8.11)). In addition, having over three employed persons dedicated to cervical cancer screening (OR = 2.34 (95%CI: 1.09–5.01)) was the factor that propels institutions to fulfil the target number. The differences in completion of colorectal cancer screening between the different characteristic groups were not significant (Table 4).

## 4. Discussion

Screenings for breast, cervical, and colorectal cancer have been incorporated into cancer prevention programs in many regions of China [11,26]. Our results show that the performance in primary healthcare institutions on colorectal cancer screening is better than in breast and cervical cancer screening. Further, more primary healthcare institutions in rural districts fulfill the target number of breast and cervical cancer screening than in urban districts. A smaller amount of staff is dedicated to breast and cervical cancer screening than to colorectal cancer screening. Therefore, an increase in the number of staff on breast and cervical cancer screening could improve cancer screening performance.

Our main finding was that the completion rates of the target number of breast and cervical cancer screening were only two-thirds of colorectal cancer screening, although, a higher uptake in the target population has been reported for breast cancer screening programs compared to colorectal cancer screening programs [11,27]. An explanation for the higher completion rate in colorectal cancer screening in our study might be that the FIT can be performed by residents themselves. For breast and cervical cancer, the commonly used CBE and pap smear tests, request to be performed by experienced doctors. Research evidence also supports the explanation that simplification of a test shows positive effects on an increased coverage and participation of screening [21,22,23].

A second explanation for the lower completion rate of breast and cervical cancer screenings is that for breast cancer screening, there is no evidence that CBE plays a role in reducing breast cancer mortality [28]. Furthermore, for cervical cancer screening, the pap smear examination has a low sensitivity for the early detection of cervical cancer in screening programs [29]. However, due to lack of equipment in the primary institutions in China, there is no alternative way to screen participants for breast and cervical cancer. This is consistent with previous works that showed that only few primary healthcare institutions can perform ultrasound, mammography, human papillomavirus testing, and thinprep cytologic tests [30,31].

The organization of the screening in Tianjin might be another explanation for low completion rates of the target number of breast and cervical cancer screenings compared to those of colorectal cancer screening. The Tianjin government has set up a specialized working group to manage and supervise the colorectal cancer screening program. However, the breast and cervical cancer screening program has been incorporated into basic public health services without independent management and supervision [32]. As a consequence, there is free access to further testing after a positive finding in colorectal cancer screening by colonoscopy, and no free access is available to further testing after positive findings in breast and cervical cancer screening. This barrier-free referral in colorectal cancer screening results in a better performance than breast and cervical cancer screening [18,33].

A next explanation might be related to the number of staff dedicated to cancer screening which was lower for breast and cervical cancer screening than for colorectal cancer screening. It has also been shown by others that the amount of staff dedicated to breast and cervical cancer is insufficient, and influences the performance of screening [30,31,34]. Moreover, primary healthcare staff in China is unevenly distributed across the country, is substantially underpaid, and often inadequately trained [24]. Apart from cancer screening, they have to perform both basic medical and basic public health services in daily work, which may lead to heavy workloads.

We observed that institutions in rural districts were more likely to fulfil the target number of breast and cervical cancer screenings than those in urban districts, where US studies report that breast and cervical cancer screening rates in urban districts are higher than in rural districts [35,36]. The main explanation for this is that in the US the main breast cancer screening tool is mammography, where in the China screening consists mainly out of CBE. Mammography capacity is limited in rural districts, and CBE does not request equipment [37]. Furthermore, research and practice have shown that cervical cancer screening rate is highly correlated with mammography screening rate [35]. Our finding is consistent with that of Yang et al., Huang et al., and Paulauskiene et al. who found that the coverage and participation of breast and cervical cancer screening were higher in rural regions compared to urban regions in China and Lithuania [38,39,40]. In addition, institutions in lower GDP districts showed a better screening performance than those in higher GDP districts. The two findings may be explained by the fact that women living in urban or high GDP districts and usually have higher education and income levels, are more likely to participate in opportunistic screening instead of population-based screening [33].

The data of our study was collected between 2017 and 2018 and reflects the cancer screening capacity of institutions in Tianjin in the last years. Because Tianjin is one of the five major cities in China, it is expected that our study results reflect the cancer screening status in other main Chinese cities that have a similar screening strategy and socioeconomic level.

This study has some limitations. First, the results and conclusions cannot be extrapolated to all of China where some regions have different screening strategies than Tianjin. Second, the data in this study relied on self-reports from institution leaders, which tended to bias. Third, we did not collect the size of the target population in the area of each institution and the total number of residents invited to screening. Thus, we did not obtain the participation and coverage rates that have been used in previous studies as screening performances. This may overestimate the screening performance, since the target number is the minimum required for an institution to provide screening.

## 5. Conclusions

Primary healthcare institutions perform better in colorectal cancer than in breast and cervical cancer screening. We identified that more staff dedicated to cervical cancer screenings could improve screening performance, and institutions in rural districts are more likely to fulfil the target number of breast and cervical cancer screenings. These findings suggest that more people should be encouraged to work in the primary healthcare system, and that the training for cancer screening should be strengthened, to augment the quantity and quality of staff dedicated to cancer screenings [34,41]. In addition, to improve population-based cancer screening performance appropriate screening techniques should be made available, more medical resources should be introduced, and program supervision should be strengthened [29,30,38]. Finally, to identify target population who are less likely to be exposed to cancer screenings is critical to screening participation and performance. This suggests that the government should provide service for low-education, rural, and the elderly populations.

## Figures and Tables

**Table 1:** The information collected in the survey.

District. Cancer screening program. Available tests and equipment. Number of medical staff dedicated to screening. Frequency of medical staff training. Frequency of introduction to residents of the screening program and knowledge. Telephone invitation of target population. Target number of screenings. Actual number of screenings. Number of suspected high-risk residents in screenings (referral rate). Fulfilling the target number of screening or not.

**Table 2:** Characteristics of all 262 primary healthcare institutions that participated in the survey on breast, cervical, and colorectal cancer screening in Tianjin, China.

Characteristics	Breast Cancer (*n* = 206)			Cervical Cancer (*n* = 231)			Colorectal Cancer (*n* = 261)		
	Category	*n* (%)		Category	*n* (%)		Category	*n* (%)	
Urban district ^†^	Yes	81	(39.3)	Yes	82	(35.5)	Yes	95	(36.4)
	No	125	(60.7)	No	149	(64.5)	No	166	(63.6)
GDP of district (¥100 million) ^‡^	≤800	105	(51.0)	≤800	126	(54.4)	≤800	137	(52.5)
	>800	101	(49.0)	>800	105	(45.5)	>800	124	(47.5)
Available screening tests	Clinical breast examination	202	(100.0)	Gynecological examination table	216	(95.6)	FIT ^§^	255	(100.0)
	Ultrasound	73	(36.1)	Pap smear test	118	(52.2)	CS ^§^	5	(2.0)
	Near-infrared scanner	11	(5.4)	Colposcope	33	(14.6)	Not reported	6	
	Mammography	0		TCT ^§^	39	(17.3)	—		
	Not reported	4		HPV-DNA test ^§^	18	(8.0)	—		
	—			Not reported	5		—		
Number of screening tests	0	0		0	10	(4.4)	0	0	
	1	125	(61.9)	1	69	(30.5)	1	250	(98.0)
	2	70	(34.7)	2	99	(43.8)	2	5	(2.0)
	≥3	7	(3.5)	≥3	48	(21.2)	≥3	0	
	Not reported	4		Not reported	5		Not reported	6	
Frequency of staff training	Once a year	182	(94.3)	Once a year	210	(95.5)	Once a year	247	(96.5)
	Less than once a year or never	11	(5.7)	Less than once a year or never	10	(4.5)	Less than once a year or never	9	(3.5)
	Not reported	13		Not reported	11		Not reported	5	
Number of staff dedicated to screening	<3	51	(26.2)	<3	50	(22.6)	<3	21	(8.3)
	3–9	113	(57.9)	3–9	134	(60.6)	3–9	53	(20.9)
	≥10	31	(15.9)	≥10	37	(16.7)	≥10	180	(70.9)
	Not reported	11		Not reported	10		Not reported	7	
Frequency of introduction to residents of screening programs	≥4 times a year	104	(54.5)	≥4 times a year	115	(52.5)	≥4 times a year	134	(52.8)
	<4 times a year	87	(45.5)	<4 times a year	104	(47.5)	<4 times a year	120	(47.2)
	Not reported	15		Not reported	12		Not reported	7	
Telephone invitation of target population	Yes	167	(88.4)	Yes	195	(89.9)	Yes	246	(96.5)
	No	22	(11.6)	No	22	(10.1)	No	9	(3.5)
	Not reported	17		Not reported	14		Not reported	6	
Target number of screenings per year	≤1000	32	(18.8)	≤1000	36	(17.9)	≤1000	41	(16.9)
	1001–2000	70	(41.2)	1001–2000	85	(42.3)	1001–2000	76	(31.4)
	>2000	68	(40.0)	>2000	80	(39.8)	>2000	125	(51.7)
	Not reported	36		Not reported	30		Not reported	19	
Number of performed screenings per year	≤1000	49	(27.8)	≤1000	57	(27.5)	≤1000	36	(14.6)
	1001–2000	77	(43.8)	1001–2000	87	(42.0)	1001–2000	78	(31.7)
	>2000	50	(28.4)	>2000	63	(30.4)	>2000	132	(53.7)
	Not reported	30		Not reported	24		Not reported	15	
Fulfilling the target number of screening or not	Yes	101	(60.5)	Yes	116	(58.6)	Yes	224	(93.3)
	No	66	(39.5)	No	82	(41.4)	No	16	(6.7)
	Not reported	39		Not reported	33		Not reported	21	
Referral rate (%)	Median (P25, P75)	1.2 (0.56, 4.15)		Median (P25, P75)	1.3 (0.48, 3.18)		Median (P25, P75)	2.2 (1.28, 4.38)	
	Not reported	84		Not reported	93		Not reported	81	

^†^ Ministry of Civil Affairs of the People’s Republic of China. http://xzqh.mca.gov.cn/ (in Chinese, accessed on 10 February 2021). Tianjin is divided into 16 districts. Urban districts: Heping, Hedong, Hexi, Nankai, Hebei, Hongqiao and Binhai. Rural districts: Dongli, Xiqing, Jinnan, Beichen, Wuqing, Baodi, Ninghe, Jinghai and Jizhou. ^‡^ Tianjin Statistical Yearbook 2018. http://stats.tj.gov.cn/nianjian/2018nj/zk/indexeh.htm (accessed on 10 February 2021). GDP: Gross Domestic Product of District. ^§^ TCT: Thinprep Cytologic Test, FIT: Fecal Immunochemical Test, CS: Colonoscopy, HPV-DNA test: Human Papillomavirus DNA test.

**Table 3:** Univariate analysis on the determinants of completion of the target number of breast, cervical, and colorectal cancer screening.

Characteristics	Category	Breast Cancer (*n* = 206)		Cervical Cancer (*n* = 231)		Colorectal Cancer (*n* = 261)	
		*n*	(%)	*n*	(%)	*n*	(%)
Urban district	Yes ^†^	27	(37.0)	25	(33.8)	80	(88.9)
	No	74	(78.7)	91	(73.4)	144	(96.0)
	OR (95%CI)	6.30 (3.18, 12.51) ***		5.41 (2.89, 10.10) ***		3.00 (1.05, 8.56) **	
	*p* value	<0.001		<0.001		0.040	
GDP of district (¥100 million)	≤800 ^†^	57	(69.5)	72	(66.1)	122	(96.1)
	>800	44	(51.8)	44	(49.4)	102	(90.3)
	OR (95%CI)	0.47 (0.25, 0.89) **		0.50 (0.28, 0.89) **		0.38(0.13, 1.13) *	
	*p* value	0.020		0.019		0.082	
Number of screening tests	<2 ^†^	54	(55.1)	32	(49.2)	214	(93.4)
	≥2	44	(67.7)	80	(62.5)	5	(100.0)
	OR (95%CI)	1.71 (0.89, 3.29) *		1.72 (0.94, 3.14) *		— ^‡^	
	*p* value	0.109		0.079		—	
Frequency of staff training	Once a year ^†^	95	(60.1)	111	(58.4)	216	(93.9)
	Less than once a year or never	5	(62.5)	4	(57.1)	6	(75.0)
	OR (95%CI)	1.11 (0.26, 4.79)		0.95 (0.21, 4.36)		0.19 (0.04, 1.05) *	
	*p* value	0.894		0.946		0.057	
Number of staff dedicated to screening ^§^	<3 ^†^	21	(46.7)	18	(40.0)	62	(89.9)
	≥3	80	(66.1)	98	(64.5)	161	(95.3)
	OR (95%CI)	2.23 (1.11, 4.47) **		2.72 (1.38, 5.39) ***		2.27 (0.79, 6.53) *	
	*p* value	0.024		0.004		0.128	
Frequency of introduction to residents of screening programs	≥4 times a year ^†^	51	(58.0)	56	(54.9)	116	(92.1)
	<4 times a year	49	(65.3)	58	(62.4)	105	(95.5)
	OR (95%CI)	1.37 (0.72, 2.58)		1.36 (0.77, 2.41)		1.81 (0.60, 5.47)	
	*p* value	0.336		0.291		0.293	
Telephone invitation of target population	Yes ^†^	88	(60.3)	103	(58.5)	214	(93.0)
	No	12	(70.6)	12	(63.2)	8	(100.0)
	OR (95%CI)	1.58 (0.53, 4.73)		1.22 (0.46, 3.24)		— ^‡^	
	*p* value	0.412		0.697		—	

* *p* < 0.2; ** *p* < 0.05; *** *p* < 0.01. ^†^ Reference. ^‡^ Not included in the regression analysis. ^§^ Cutoff value of the number of staff dedicated to screening colorectal cancer screening is: <10 staff members (reference), ≥10 staff members.

**Table 4:** Multivariate analysis on the determinants of completion of the target number of breast, cervical, and colorectal cancer screening.

Characteristics	Category	Breast Cancer	Cervical Cancer	Colorectal Cancer
		OR (95%CI)	OR (95%CI)	OR (95%CI)
Urban district	Yes ^†^	1	1	1
	No	5.16 (2.51, 10.63) **	4.17 (2.14, 8.11) **	2.90 (0.91, 9.26)
GDP of district (¥100 million)	≤800 ^†^	1	1	1
	>800	0.63 (0.31, 1.29)	0.78 (0.40, 1.48)	0.49 (0.15, 1.56)
Number of screening tests	<2 ^†^	1	1	— ^‡^
	≥2	1.35 (0.65, 2.81)	1.44 (0.74, 2.81)	—
Number of staff dedicated to screening	<3 ^†^	1	1	1 ^§^
	≥3	1.54 (0.69, 3.43)	2.34 (1.09, 5.01) *	2.90 (0.93, 9.05)
Frequency of staff training	Once a year ^†^	— ^‡^	— ^‡^	1
	Less than once a year or never	—	—	0.21 (0.03, 1.26)

* *p* < 0.05; ** *p* < 0.001. ^†^ Reference. ^‡^ Not included in the regression analysis. ^§^ Cutoff value of the number of staff dedicated to screening colorectal cancer screening is: <10 staff members (reference), ≥10 staff members.

## Data Availability

The data presented in this study are available on request from the corresponding author. The data are not publicly available because other studies related to the project are ongoing.

## Supplementary Materials

The following are available online at https://www.mdpi.com/1660-4601/18/6/3312/s1, Table S1: Univariate analysis on the determinants of completion of the target number of breast, cervical, and colorectal cancer screening in urban district, Table S2: Univariate analysis on the determinants of completion of the target number of breast, cervical, and colorectal cancer screening in rural district.
