# Supplementary material for: Determinants of Population-Based Cancer Screening Performance at Primary Healthcare Institutions in China
Source: Int J Environ Res Public Health. 2021 Mar 23;18(6):3312. doi: 10.3390/ijerph18063312 (PMC8005057; doi:10.3390/ijerph18063312)
Supplement: Supplementary file 1 [file ijerph-18-03312-s001.pdf]

Supplementary Materials

Univariate analysis stratified by urban and rural

Table S1. Univariate analysis on the determinants of completion of the target number of breast, cervical, and colorectal cancer screening in urban district

Characteristics	Category	Breast cancer (n=81)	Cervical cancer (n=82)	Colorectal cancer (n=95)
		OR(95%CI)	OR(95%CI)	OR(95%CI)
GDP of district (￥100 million)	≤800†	1	1	1
	>800	0.67(0.25, 1.76)	0.53(0.20, 1.41)	0.18(0.02, 1.46)*
Number of screening tests	<2†	1	1	—‡
	≥2	1.27(0.46, 3.54)	0.96 (0.36, 2.52)	—
Frequency of staff training	Once a year†	1	1	1
	Less than once a year or never	1.15(0.18, 7.34)	1.33 (0.21, 8.55)	0.23(0.02, 2.81)
Number of staff dedicated to screening§	<3†	1	1	1
	≥3	1.74(0.63, 4.79)	2.08(0.70, 6.14)*	6.98(1.64, 29.79)**
Frequency of introduction to residents of screening programs	≥4 times a year†	1	1	1
	<4 times a year	1.52(0.56, 4.11)	1.13 (0.41, 3.10)	1.19(0.29, 4.97)
Telephone invitation of target population	Yes†	1	1	—‡
	No	0.82(0.14, 4.81)	0.36(0.04, 3.25)	—

* *P*<0.2; ***P*<0.01. † Reference. ‡ Not included in the regression analysis. § Cutoff value of the number of staff dedicated to screening colorectal cancer screening is: ＜10 staff members (reference), ≥10 staff members.

Table S2. Univariate analysis on the determinants of completion of the target number of breast, cervical, and colorectal cancer screening in rural district

Characteristics	Category	Breast cancer (n=81)	Cervical cancer (n=82)	Colorectal cancer (n=95)
		OR(95%CI)	OR(95%CI)	OR(95%CI)
GDP of district (￥100 million)	≤800†	1	1	1
	>800	0.53 (0.20, 1.43)	0.90(0.39, 2.08)	1.17(0.21, 6.58)
Number of screening tests	<2†	1	1	—‡
	≥2	1.34(0.48, 3.72)	1.89 (0.79, 4.49)*	—
Frequency of staff training	Once a year†	1	1	1
	Less than once a year or never	—	—	0.15(0.01, 1.54)*
Number of staff dedicated to screening§	<3†	1	1	1
	≥3	1.56 (0.48, 5.07)	2.11(0.77, 5.73)*	0.94(0.17, 5.31)
Frequency of introduction to residents of screening programs	≥4 times a year†	1	1	1
	<4 times a year	0.54(0.18, 1.60)	0.90 (0.40, 2.02)	1.86(0.30, 11.46)
Telephone invitation of target population	Yes†	1	1	—‡
	No	2.70(0.32, 22.58)	2.09(0.44, 9.98)	—

* *P*<0.2. † Reference. ‡ Not included in the regression analysis. § Cutoff value of the number of staff dedicated to screening colorectal cancer screening is: ＜10 staff members (reference), ≥10 staff members.
